# Medi-Cinema: A Pilot Study on Cinematherapy and Cancer as A New Psychological Approach on 30 Gynecological Oncological Patients

**DOI:** 10.3390/cancers14133067

**Published:** 2022-06-22

**Authors:** Daniela Pia Rosaria Chieffo, Letizia Lafuenti, Ludovica Mastrilli, Rebecca De Paola, Sofia Vannuccini, Marina Morra, Fulvia Salvi, Ivo Boškoski, Vanda Salutari, Gabriella Ferrandina, Giovanni Scambia

**Affiliations:** 1 Clinical Psychology Unit, Fondazione Policlinico Universitario Agostino Gemelli IRCCS, 00168 Rome, Italy; danielapiarosaria.chieffo@policlinicogemelli.it (D.P.R.C.); ludovica.mastrilli@guest.policlinicogemelli.it (L.M.); depaola.rebecca@gmail.com (R.D.P.); vannuccini.sofia@gmail.com (S.V.); 2 Medicine and Surgery Faculty, Catholic University of the Sacred Heart, 00168 Rome, Italy; giovanni.scambia@policlinicogemelli.it; 3 Gynecologic Oncology Unit, Women Wealth Area, Department of Woman and Child Health and Public Health, Fondazione Policlinico Universitario Agostino Gemelli IRCCS, 00168 Rome, Italy; vanda.salutari@policlinicogemelli.it (V.S.); mariagabriella.ferrandina@policlinicogemelli.it (G.F.); 4 Medicinema Italia Onlus, 20122 Milan, Italy; m.morra@medicinema-italia.org (M.M.); f.salvi@medicinema-italia.org (F.S.); 5 Centre for Endoscopic Research Therapeutics and Training (CERTT), Università Cattolica del Sacro Cuore, Fondazione Policlinico Universitario Agostino Gemelli IRCCS, 00168 Rome, Italy; ivo.boskoski@unicatt.it; 6 Department of Life Sciences and Public Health, Università Cattolica del Sacro Cuore, 00168 Rome, Italy

**Keywords:** cinema, cancer, depression, female tumor, gynecologic oncology, group therapy, hospitalization, COVID-19 pandemic, cinematherapy, patient-centered care

## Abstract

**Simple Summary:**

During the COVID-19 pandemic, as clinical psychologists, we felt a human need—in addition to an ethical duty—to guarantee continuity of care to our patients, many of whom felt alone and frightened or verbalized the fear of being abandoned. This provided a channel of psychological support that helped patients to face, together, such a difficult moment. In consideration of the difficulty of accessing hospitals during this period, and the fear felt by many patients of face-to-face visits for psychological interviews, we devised a sharing project that could be used remotely. In so doing, we responded to the need to share and normalize that we observed in our patients, with this pilot project aiming at evaluating the effectiveness of cinema as a tool for emotional mediation in psychological support paths.

**Abstract:**

Background: Several subjects affected by cancer experience a significant level of multidimensional disease. This longitudinal study aims to evaluate the effectiveness of psycho-oncological support using Cinema as an emotional mediator and to promote perceived well-being by personalized psychological treatment. Methods: Thirty women diagnosed with gynecological cancer watched 12 movies and participated in a psychotherapy group co-conducted by two psychotherapists. Patients completed nine questionnaires at T0 (baseline), T1 (3 months) and T2 (6 months). Results: Patients observed significant improvements (CORE-OM: *p* < 0.001) in psychological well-being. The results showed statistically significant differences, even in several other dimensions, such as Anxiety (STAY-Y1-2: *p* < 0.001), Empathy (BEES, *p* < 0.001), Coping (COPE: *p* < 0.001), QoL (QLQ-C30, *p*: 0.026), couple relationship (DAS, Satisfaction: *p*: 0.013; Cohesion: *p*: 0.004) and alexithymia (TAS-20, Difficulty Identifying Feeling: *p*: 0.002; Externally-Oriented Thinking: *p*: 0.003). Conclusions: The data show that cinema, as an innovative psychological approach, could be a valid instrument to support patients in oncological pathways as well as facilitating the process of recognizing themselves in other patients and communicating about their own feelings.

## 1. Introduction

Whatever the diagnosis and prognosis, cancer is always significant. In fact, cancer represents, for patients and their families, an overwhelming existential experience that concerns multiple aspects of life: the meaning given to suffering, illness, death, as well as body image and family, social, and professional relationships. Individuals suffering from cancer may experience a broad spectrum of physical and psychological disorders related to the different phases of treatments and the adverse effects of specific therapies. Observational data show that, according to the studied population, 20–60% of subjects affected by cancer experience a significant level of multidimensional disease. On average, about 35–40% of people with cancer have a psychiatric disorder that can be classified according to ICD-10 or DSM-5 (major depression, dysthymia, anxiety, adaptation). Psychopathological disorders, in specific depressive conditions, are independent risk factors of several potential negative outcomes: worsening of quality of life; increased risk of psychological distress in the family; reduced adherence to treatments; alteration of the doctor–patient relationship; and development of “abnormal illness behavior”. In oncology, a systemic approach called P4 medicine has been proposed (Predictive, Preventative, Personalized, Participatory). This approach is an extension of what is usually called personalized or genomic medicine [1]. P4 medicine creates effective predictive, personalized, and preventive action and collaborative models for treatments. Based on the a study conducted with gynecological oncological patients [2], we believe that these types of cancers require multidimensional and focused interventions, since patients have complex, specific, and unmet needs. Such a personalized approach also integrates psycho-cognitive aspects in order to empower the patient, increase her quality of life, and transform her from a passive into an active decision maker in the treatment process [3]. With gynecological oncological diseases, patients feel a sense of loss linked not only to the lowering of mood or to the sense of psychophysical loss, but also—and above all—to the perception of the loss of female identity, physical integrity, decreased libido, premature menopause, infertility, changes in couple life, weight-related matters, and social isolation. Gynecological cancers mainly affect the uterus (endometrium and cervix) and ovaries. They lead to different prognoses and paths, and consequently have different psychological impacts. Because of its severity, these kinds of cancer are accompanied by different psychological and emotional problems which almost 79% of patients find difficult and embarrassing to express to their oncologist, such as the fear of illness and treatments and the inevitable consequences of therapy on body image, self-esteem, sexuality, and romantic relationships [4]. A psycho-emotional burden accompanies women throughout the diagnostic, therapeutic process, and beyond. Patients have to accept physical changes, a different QoL, their new balance, and their different needs. During this period, supportive clinical psychological interventions are necessary to accompany the woman through the adaptation process, which is not always linear and simple.

A previous study [5] demonstrated the effectiveness of psychological support in patients with cancer, showing a significant decrease in distress, anxiety, and depression. Since cancer is a systemic disease, the only way to effectively fight it is through a multidimensional approach. The history of each patient is unique and must be reconstructed by a multidisciplinary team, considering not only the organic component but also the psychological one. The oncological experience requires attention to emotional, clinical, and social needs in the form of constant monitoring and personalized interventions. Different studies have shown that cancer can severely impair a woman’s identity, self-esteem, body image, and intimacy [6]. That is the reason why clinics have to offer personalized psychological treatment which takes into account both the pathology and the patient, including her emotional experiences. Psychological suffering ought to be considered a vital parameter like physical pain. Personalized psycho-oncological interventions could provide, for the patient and her family, a better quality of life, solid support during the illness path, and an enhanced ability to manage emotions.

During the COVID-19 pandemic, several categories of patients—such as oncologic patients—felt particularly exposed to the risks of the disease. This generated isolation, an emotional loneliness in contrast with the need to communicate, to share, to feel less alone. Life plans were temporarily put on hold. For this reason, we created a project that could build a bridge between these patients in order to help them share both their pain and successes, motivating them to avoid isolation and to look at each other, discovering emotions that can make them feel stronger. In this sense, cinema was not considered a therapy per se, but rather, a complementary strategy and tool in addition to traditional psychological practice that could reach and involve patients in a multidimensional way (sensorial, emotional, organic, linguistic, interpersonal) [7]. Dr. G. Solomon defined the term ‘Cinematherapy’ as the use of movies as therapy, stating that many psychological discomforts could be managed and improved by watching specific films. Analyzing about 200 films assigned to different kinds of patients, he demonstrated the therapeutic properties of cinema as a support in the therapeutic process. Cinematherapy can be a powerful healing and growth tool for anyone who is open to learning and watching movies with awareness and attention. Cinematherapy allows us to use every aspect of the film (such as images, plot, music, etc.) to gain understanding, inspiration, emotional release or relief, and natural change [8]. Films in therapy can be a support for questioning maladaptive beliefs; they allow people to see behavior from an external point of view. Identifying with a character in a film can help people to rediscover their individual resources that are sometimes inaccessible on a rational level, gaining a greater awareness of their own tools and strategies for coping with certain situations and difficulties. Cinema therapy involves seven areas of functioning: logical (plot development), linguistic (dialogues), visual-spatial (images, colors, symbols), musical (sounds and music), interpersonal (storytelling and relating), kinaesthetic (movement), and intrapsychic (internal functioning). In addition, films amplify emotions and allow those who watch them to get in touch with their feelings and experience new and desired behaviors. Sharing within a group the feelings and emotions experienced while watching a film allows patients to explore and acknowledge their emotions in a protected context and to process them with greater awareness. Films viewed at home allow have the potential to serve as bridges that can integrate aspects related to therapy and those inherent in their own lives.

As B. Wolz wrote [9] that there is a fundamental link between film narration and introspection. The story has a transformative and healing effect on the human mind, and therefore, can prove to be a very useful tool in individual and group therapeutic paths. F. Ulus [10] proposed the use of excerpts rather than entire films in his studies, stating that this methodology can provide patients with the three Es: Entertainment (i.e., a comfortable atmosphere during a treatment setting); Education (information); and Empowerment (motivating patients). However, this method seems to have been more effective with patients who had been administered high doses of drugs, for whom certain movie scenes were a useful tool to motivate them to understand each other better (through the process of normalization, sharing, and understanding their symptoms), and therefore, to increase compliance with the therapeutic process [11].

As described by Dermer and Hutchings [12], it is possible to approach cinematherapy interventions in three steps: (1) Assessment: the therapist chooses a film based on its inclusion of a specific issue; (2) Implementation: the viewing of the film; (3) Explanation: discussion about the film. Other authors [13] identified four steps: (1) Identification: the patient identifies with a specific character; (2) Categorization: the patient tries to learn from his/her own experiences; (3) Insight: the patient internalizes the experience of the character and increases his/her self-awareness; and (4) Universalization: the patient understands that having a common experience allows him/her to not feel alone.

Cinema is an experimental laboratory in which cognitive and affective processes can be stimulated and/or reassembled. Cinema allows people to dream, satisfy unexpressed desires, identify with characters, and project unconscious experiences, thereby having cathartic and suggestive effects. As such, cinema-therapy is a form of complementary medicine which be used to treat and support patients. The therapeutic group itself represents a powerful emotional and cognitive tool for individual and institutional work and learning. Within groups, we observe how cohesion and acceptance allow patients to discover that suffering is something that can be shared.

**Figure A1:**
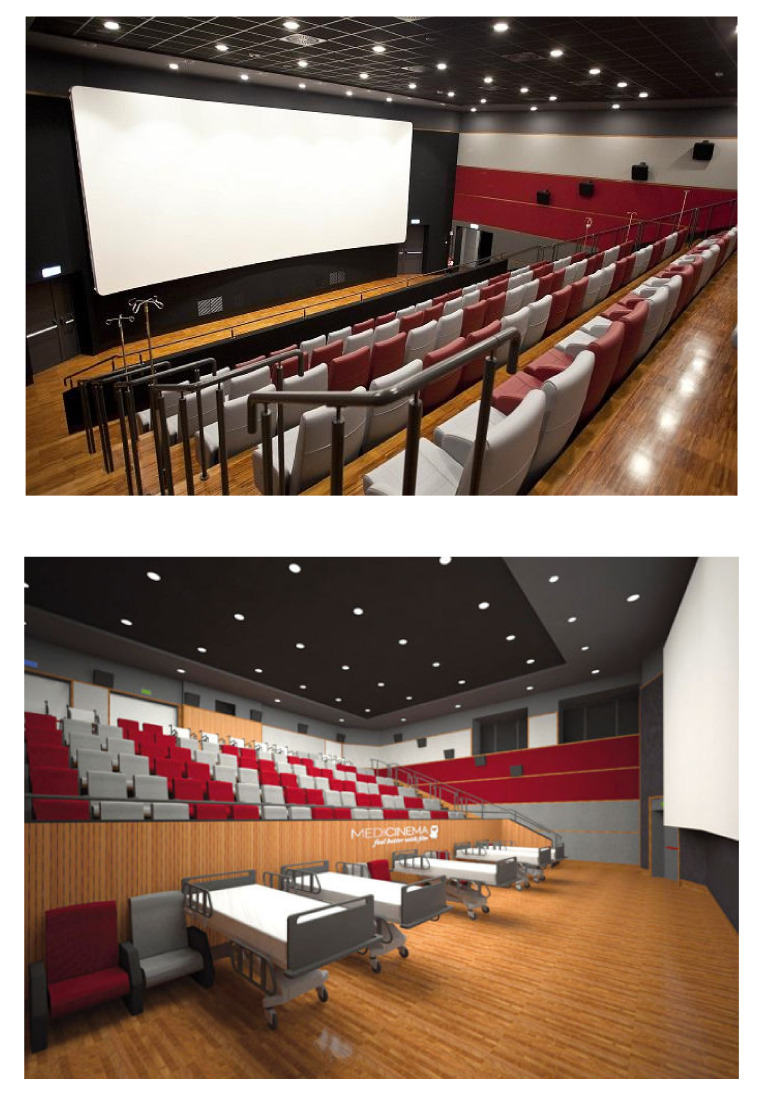
Medicinema Hall at Policlinico Agostino Gemelli, Rome.

Female organic pathologies (such as gynecological cancer) are complex, and treatments have to consider not only the physical and biological implications, but also, and perhaps most importantly, the psychological effects on patients, e.g., psychological distress, lifestyle changes, the occurrence of fears and anxieties, and finally, the sense of being a woman in a new psychological dimension. “Medicinema” (a portemanteau of “medicina” and “cinema”) is aimed to encourage a process of re-construction of this dimension using filmic images. MediCinema Italia—The Cinema That Cures [14] is a non-profit organization founded in 2013 and inspired by MediCinema UK, an organization that has been active in Great Britain since 1996. This is the first project at the Italian national level that aims to bring culture and entertainment to hospitals for therapeutic purposes (Figure A1).

## 2. Materials and Methods

A prospective observational study was conducted at the Female Tumors Day Hospital and at the Gynecological Oncological ward of Fondazione Policlinico Universitario A. Gemelli IRCCS.

The project was developed in collaboration with “Medicinema Italia”, which provided the distribution of the selected films. Patients were able to watch these films remotely through online platforms, given that the COVID-19 pandemic did not allow the viewing of films in the cinema at the Policlinico Gemelli. From May to October 2021, 30 women with gynecological cancer were enrolled in this project.

Patients were asked to watch 12 films chosen by the authors (clinical psychologists), i.e., two per month (see the program in Appendix A, Table A1 and Table A2), and to participate in group therapy conducted by two psychotherapists of the Clinical Psychology Unit of the Fondazione Policlinico Universitario A. Gemelli IRCCS. The meeting was structured as follows: opening and greetings, free comments on the medinn film viewed, free sharing of emotional aspects and/or personal experiences related to the film or particular scenes, feedback from psychotherapists regarding the choice of the film, a free discussion concerning particular thematic areas, and final restitution by the therapists.

The selection of the 12 films was made by the authors based on a collaboration with Medicinema, which made available to the project the entire filmography of the most famous online platforms (Infinity, Chili) as well as relevant technical data sheets. Medicinema also allowed participants to preview movies. The films were subsequently selected based on the following criteria:

- a focus on the issues that most frequently emerge in the course of psychological work with oncological gynecology patients;
- the way in which these issues are explored and dealt within the films.

The main objective was to expose patients to various types of films (drama, comedies, biographies, etc.) in order to give them the opportunity to identify themselves from different perspectives and to consider aspects of daily life not only linked to illness, but also to gradual recovery.

Patients completed questionnaires at T0 (baseline), T1 (3 months), and T2 (6 months).

### 2.1. Participants

Thirty patients affected by gynecological cancer were enrolled. Enrollments were carried out on the basis of the following inclusion and exclusion criteria:

Inclusion criteria:
  - Patients affected by gynecological cancer (ovary, cervix, endometrium)
  - Aged ≥18 years
  - Patients undergoing active oncological treatments (surgery, chemotherapy, maintenance treatments or other)
  - Presence at the Oncological Gynecology ward or Female Cancer Day Hospital
  - Able to understand and provide informed consent
Exclusion criteria:
  - Patients in comorbidity with other psychiatric pathologies
  - Low adherence to care and/or reduced compliance
  - Aged >70 years
  - Not undergoing oncological treatments
  - Inability or unwillingness to provide informed consent
  - Patients with preexisting psychopathological disorders
  - Patients affected by severe language deficits
Of the entire enrolled group (30 patients):
  - 5 did not complete the test correctly and/or did not participate at the first group meeting: during phone interviews with these patients to determine the reasons/difficulties, we found that 3 had experienced disease progression and felt disinclined to participate in this kind of project, while 2 decided to continue oncological therapy in their respective regions and dropped out.
  - 2 patients completed only the T0 test (they died) and were not included in our analysis.
  - 23 patients completed just T0 and T1 tests, but not T2 (2 of them died and 2 dropped out after T1 for personal reasons).
  - The remaining 19 patients completed T0, T1, and T2.

### 2.2. Questionnaires

All patients were asked to complete the following self-administered questionnaires at T0 (baseline), T1 (3 months), and T2 (6 months):

- Toronto Alexithymia Scale (TAS-20) [15,16]: to evaluate alexithymia, i.e., people who have difficulties identifying and describing emotions and tend to minimize emotional experiences, instead focusing attention externally.
- State-Trait Anxiety Inventory (STAI-Y) [17]: to assess the trait and state anxiety domains.
- General Self Efficacy (GSE) [18]: a self-reporting tool consisting of 10 items, intended to measure self-efficacy. It aims to assess the beliefs a person has about his/her ability to cope with a variety of daily life demands.
- Symptom Checklist-90-R (SCL-90-R) [19,20]: to evaluate psychopathological symptoms. A self-reported, 90-item psychometric tool that objectively evaluates a broad range of symptoms of psychopathology. It measures nine symptom dimensions, i.e., somatization, interpersonal sensitivity, obsessive-compulsive symptoms, depression, anxiety, hostility, phobic anxiety, paranoid ideation, and psychoticism, as well as a class of additional items that assess other aspects. It can provide an overview of a patient’s psychological symptoms and their intensity at a given time point.
- The European Organization for Research and Treatment of Cancer Quality of Life Questionnaire-C30 (EORTC QLQ-C30) [21]: Quality of life questionnaire, specifically tailored for cancer samples. It consists of 30 Likert scale items that satisfy three factors: (1) physical assessment; (2) emotional assessment; and (3) fatigue assessment.
- Dyadic Adjustment Scale (DAS) [22]: to assess the quality of romantic relationships. This tool evaluates the adaptability, quality, and representation that each partner has with regard to an intimate relationship. It is a self-reporting questionnaire consisting of 32 items, divided into four subscales: dyadic satisfaction, dyadic cohesive, dyadic and affective consent.
- Clinical Outcomes in Routine Evaluation (CORE-OM) [23,24]: a self-administered questionnaire with 34 items to evaluate the outcome of psychological interventions. Each item refers to the last week and is evaluated on a five-point scale (from never to very often or always). The CORE items refer to four domains: subjective well-being (4 items), symptoms/problems (12 items), functioning (12 items), and risk (6 items).
- Coping Orientation to Problems Experienced (COPE) [25]: A self-reporting questionnaire used to measure a person’s ability to cope with stressful situations. The assessment of this ability is related to some variables of a psychosocial nature, expressed in items that consider the dispositional and situational aspect that determine reactions to stressful events.
- Balanced Emotional Empathy Scale (BEES) [26]: a self-assessment questionnaire consisting of 30-items that measure an individual’s ability to empathize with the emotional experiences of others.

The choice to administer nine scales was dictated by the desire to investigate different psychological dimensions.

### 2.3. Statistical Analysis

The statistical procedures involved in the identification of the sample and the acceptance/refutation of the research hypotheses were carried out using the IBM SPSS Statistics 28 (Statistical Package for Social Science) data analysis software. For sample identification, descriptive statistics (mean and ds) and frequency analyses of sociodemographic variables were performed. For the detection of significant differences (*p* < 0.05) between the surveys analyzed at two time points, a paired-samples T Test was used. In addition, the repeated-measures ANOVA test was used to detect significant differences (*p* < 0.05) between the surveys analyzed at three separate time points.

## 3. Results

The sample of 30 patients comprised woman aged from 23 to 70 years, with a mean age of 49.37 (standard deviation: 10.15). Additionally, 73.3% of the sample was employed, while the remaining 26.7% did not have an occupation.

Statistical analyses were conducted on two samples (Table 1):

(a) the sample of all patients who completed the questionnaires at T0 and T1 (23 patients)
(b) the sample of 19 patients who also completed the questionnaires at T2.

Sample (a) consisted of 23 women ranging from 23 to 70 years of age, with a mean age of 50.39 years and a standard deviation (ds) of 10.78. The majority of the sample (73.9%) was employed.

Sample (b) consisted of 19 women with a mean age of 49.16 years (ranging from 23 to 70) and a standard deviation (ds) of 11.03. The majority of the sample (68.4%) was employed.

### 3.1. t-Test with Paired Samples for Time 0 (T0) and Time 1 (T1): Sample of 23 Subjects

The results showed a statistically significant difference in several areas between the T0 and T1 assessments.

**Figure 1:**
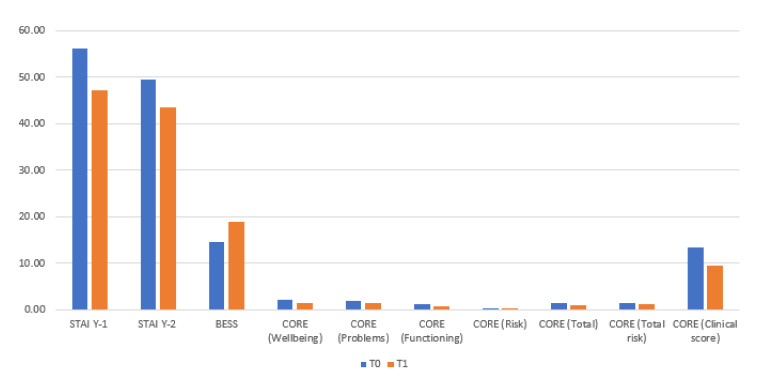
*t*-test with paired samples for T0 and T1: significant changes.

As shown in Table 2 and Figure 1, a significant decrease was found of the CORE scale and all subscales: CORE Wellbeing (*p* < 0.001); CORE problems (*p* = 0.002); CORE functioning (*p* < 0.001); CORE risk (*p* = 0.026); CORE total (*p* < 0.001); CORE total-risk (*p* = 0.003); CORE clinical score (*p* < 0.001). A significant decrease can be observed in the STAI Y-1 (state anxiety) scale scores (*p* = 0.002) and in the STAI Y-2 (trait anxiety) scale (*p* < 0.001). Finally, a significant increase in scores can be observed in the Empathy scale (BEES) (*p* = 0.002).

### 3.2. Repeated-Measures ANOVA for Time 0 (T0), Time 1 (T1), and Time (T2): Sample of 19 Subjects

**Figure 2:**
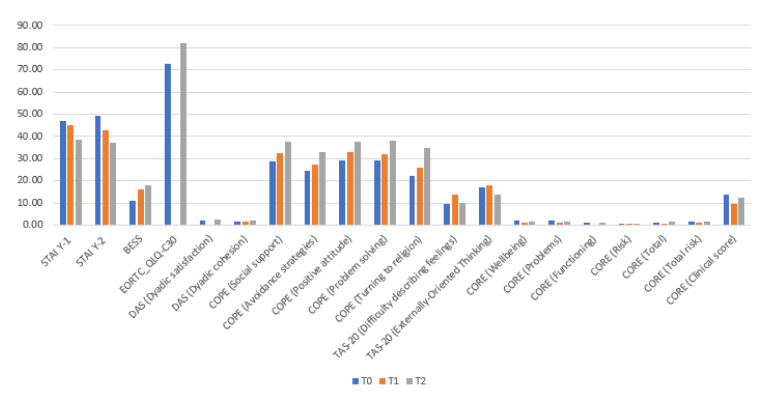
Repeated-Measures ANOVA for T0, T1, T2: significative changes.

Table 3 and Figure 2 reports the results obtained from the comparison made between the three test times. The results show that there was a significant difference between the detections made at the three different moments of the STAI scale, both in the Y-1 subscale (state anxiety) (*p* < 0.001) and the Y-2 subscale (trait anxiety) (*p* < 0.001), as well as in the Empathy scale (BEES) (*p* < 0.001).

Statistically significant differences were found in the EORTC QLQ-C30 (quality of life) (*p* = 0.026).

On the DAS scale, statistically significant differences were found in the two subscales: Dyadic Satisfaction (*p* = 0.013) and Dyadic cohesion (*p* = 0.004). No significant changes were observed in the other two subscales.

On the COPE scale, analyses revealed statistically significant differences in the subscales: Social support (*p* < 0.001), Avoidance strategies (*p* < 0.001), Positive attitude (*p* < 0.001), Problem solving (*p* < 0.001), and Turning to religion (*p* < 0.001).

The results showed that there was a significant difference between the readings taken at the three different times of the TAS-20 scale in the subscales: Difficulty Identifying Feelings (*p* = 0.002) and Externally-Oriented Thinking (*p* = 0.003); however, no difference was found in the Difficulty Describing Feelings (*p* = 0.060).

Finally, statistically significant differences were found in the subscales of the CORE test: Wellbeing (*p* < 0.001), Problems (*p* = 0.004), Operation (*p* < 0.001), Risk (*p* < 0.001), Total (*p* < 0.001), Total-Risk (*p* < 0.001), and Clinical score (*p* < 0.001).

## 4. Discussion

This study aimed to investigate the effectiveness of cinema as an emotional mediator, together with a patient group therapy, in the elaboration process of the oncological treatment pathway in women with gynecological cancer. To achieve this goal, we used a psychodiagnostic assessment tool administered at baseline (T0), after 3 months (T1), and after 6 months (T2). Our first hypothesis was that watching movies dealing with specific issues, together with the psychological support provided in group therapies the day after viewing the film, could help patients to process their oncological disease and manage the psychological difficulties related to it.

A study by Dumtrache [27] showed the importance of the use of cinema as a support in the “... personal development process, in the modeling of emotional, value and behavioral dimensions of human personality”. Another study by Batubara [28] demonstrated an increase in the QoL aspects with the use of cinematherapy-based interventions (*p*-value < 0.001). In a review of 453 articles from 1974 to 2018 concerning the use of cinema and video as supportive instruments [28], a positive outcome was found with classical cinematherapy. In line with these findings, our study showed a significant increase in the QoL area measured by EORTC (QLQ-C30).

The same article by Sacilotto [29] showed that films help couples to become more conscious about their problems and speak more positively about them. In line with those studies, our research confirmed that patients experiment with significant changes in dyadic satisfaction and cohesion subscales of the DAS test. One explanation for this is that not only do some of the films deal with relevant themes, allowing patients to elaborate on them in the therapy group, but also that, during the pandemic, the patients told us that they had watched such films together with their partners and were able to discuss such topics.

According to a study by Kim [30] that evaluated the effects of cinema-based therapy on depression and ego integrity among elderly people in a nursing home, this kind of support plays a determinant role in reducing depression and increasing a sense of balance. In line with this, in general, thanks to the results obtained in the CORE scale, our study demonstrated the effectiveness of the psychological path, through group therapy and the use of cinema as an emotional mediator, in the elaboration of the oncological treatment path. In fact, all subscales of the CORE test showed a significant decrease. This study also confirmed the effectiveness of personalized psychological support with cinema as an emotional mediator and activator. In addition, during the same period, patients had to contend with the Covid pandemic, and so felt more vulnerable and communicating about their emotions and feelings was more complicated. For that reason, it was important to promote a project that could allow patients to find themselves in other women, to feel less isolated, and to share their personal stories. In fact, our results show that patients obtained statistically higher scores from T0 to T2 in the BEES scales, confirming the hypothesis that discussing common themes and issues can help patients recognize and express emotions in empathy with others. Our results show that every kind of emotion, even the negative ones that may emerge under such circumstances, could be shared in the group and welcomed by other patients, facilitating the recognition and expression of experiences and feelings. Moreover, considering emotions, our study demonstrated that cinema—associated with psychological therapy—can help patients to better identify feelings, as seen in the results of TAS-20 administration.

Another relevant finding from our study was a significant and progressive decrease in state and trait anxiety levels, a symptom of a good process of elaboration. A recent study by Testoni [31] suggested that Cinema has been an important tool for helping people to cope with the COVID-19 Pandemic. In line with these results, our study showed that personalized psychological support with cinema improved patients’ coping strategies. Through the use of cinema, patients exhibited enhanced ability to cope with stressful situations, such as COVID-19 and cancer.

Our experience with “Medicinema” led us to develop an innovative approach to caring for patients with gynecological cancer. Psychological aspects are unique; therefore, it is essential to treat each person/patient individually. Considering this psychological unicity, decisions about treatments must be based upon aspects of personalized psycho-cognitive treatments. The patient is not only a biological and genetic entity, but a person with specific needs, values, habits, behaviors, hopes, fears, beliefs, and cognitive dispositions [32].

No patient complained of an excessive workload for any of the nine proposed scales; indeed, all tests are of reasonable duration and easy to complete. The same test battery could therefore be re-proposed in future studies. 

## 5. Conclusions and Future Perspectives

In conclusion, these results suggest that the incorporation of an innovative psychological approach—such as cinema as an emotional mediator—to routine psychological support measures may be important in terms of helping patients cope with and process the psychological distress resulting from the oncological care that they are receiving. On the basis of these results, in the future, we would like to compare innovative psychology pathways that use different emotional mediators in order to evaluate which is the most effective for cancer patients.

Considering that the project was carried out during the COVID-19 pandemic, it is possible to imagine future developments of this pilot study in the form of a selection of films that could support patients in terms of their own empowerment and post-traumatic growth with respect to the effects of the pandemic.

### Study Limitations

As a pilot study, inevitably, a number of limitations were present in this research. One was certainly a result of the COVID-19 pandemic, due to which it was not possible to watch the films together; rather, each patient had to do it individually. This certainly contributed to improving the relationship of the couple who shared this emotional experience, but at the same time, it may have diminished the emotional impact of a shared vision experience. The pandemic gave rise to another limitation: our study period was from May 2021 to October 2021, so although more than one year had passed since the beginning of the pandemic, we cannot exclude the possibility that patients’ baseline psychological statuses were affected by it.

In our clinical experience, we have realized that in the cancer treatment pathway, disease progression (understood as improvement or worsening) influences psychological aspects; this inevitably created a limitation in our research, namely, all patients were enrolled and investigated during active treatment (e.g., chemotherapy). Although we tried to enroll a homogeneous group, every oncological pathway is different, and as such, it is not possible to exclude the possibility that some patients experience improved psychological well-being as a result of a better oncological outcome.

Furthermore, this pilot study was conducted on a sample provided by clinicians at the Day Hospital on the basis of standard described criteria (patients over 70 were excluded because they are less predisposed to using technology to view movies independently at home and less willing to travel to the polyclinic for group meetings). Therefore, it was not possible to obtain a large, homogeneous sample. In addition, the risk of bias, excessive dropout, and the lack of a control group led us to create no distinction in terms of the difference between the exposure to a certain film and the factor linked to a given group. As such, future research with more specific sampling is needed.

Finally, taking into account the aforementioned limitations, two ongoing studies will also focus on the use of the EORTC CX24 and OV28 modules, providing an evaluation on the use of cinema, writing, and photography as therapeutic tools.

A final word on our statistical methods: given the large number of variables investigated in the course of this study, it seems necessary to include the presence of potential randomness among the limitations. In order to further confirm the collected data, it would be appropriate to continue this research by administering the battery of tests presented herein by implementing statistical analyses. 

## Figures and Tables

**Table 1:** Characteristics of the sample.

Sample	Entire Group (*n* = 30)	Sample T0 (*n* = 23)	Sample T1 (*n* = 23)	Sample T2 (*n* = 19)
Age M (DS)	49.37	50.39	50.39	49.16
	(10,152)	(10,782)	(10,782)	(11,032)
Employers N (%)	22	17	17	13
	(73.3%)	(73.9%)	(73.9%)	(68.4%)
Non-employees N (%)	8	6	6	6
	(26.7%)	(26.1%)	(26.1%)	(31.6%)

**Table 2:** *t*-test for paired sample at T0-T1 (sample of 23 subject).

Questionnaires	M	DS	*t*	gl	*p* Value
STAI Y1_State Anxiety	9.04	12.15	3.57	22.00	0.00
STAI Y2_Trait Anxiety	6.00	7.21	3.99	22.00	<0.001
BEES	−4.26	5.81	−3.52	22.00	0.00
EORTC_QLQ-C30	−4.73	17.91	−1.27	22.00	0.22
DAS_Dyadic consensus	0.15	1.04	0.70	22.00	0.49
DAS_Dyadic satisfaction	0.19	0.96	0.93	22.00	0.37
DAS_Dyadic cohesion	0.12	0.74	0.76	22.00	0.45
DAS_Affectional expression	0.19	0.89	1.01	22.00	0.32
SELF EFFICACY	1.57	5.44	1.38	22.00	0.18
COPE_Social support	0.17	11.56	0.07	22.00	0.94
COPE_Avoidance strategies	0.17	9.40	0.09	22.00	0.93
COPE_Positive attitude	0.39	12.75	0.15	22.00	0.88
COPE_Problem solving	0.96	11.46	0.40	22.00	0.69
COPE_Turning to religion	−0.78	10.26	−0.37	22.00	0.72
TAS-20_Difficulty Describing Feelings	−1.00	13.36	−0.36	22.00	0.72
TAS-20_Difficulty Identifying Feeling	−1.70	7.98	−1.02	22.00	0.32
TAS-20_Externally-Oriented Thinking	2.09	9.27	1.08	22.00	0.29
CORE_Wellbeing	0.72	0.55	6.29	22.00	<0.001
CORE_Problems	0.58	0.80	3.50	22.00	0.00
CORE_Functioning	0.52	0.54	4.60	22.00	<0.001
CORE_Risk	0.15	0.30	2.40	22.00	0.03
CORE_Total	0.40	0.38	5.05	22.00	<0.001
CORE_Total_Risk	0.36	0.51	3.36	22.00	0.00
CORE_Clinical Score	3.80	3.63	5.03	22.00	<0.001

**Table 3:** Repeated-Measures ANOVA for T0, T1, T2: sample of 19 subjects.

Questionnaires	F	gl	*p* Value	h^2^
STAI Y1_State anxiety	23,203	2–36	<0.001	0.563
STAI Y2_Trait Anxiety	22,964	1359–24,463	<0.001	0.561
BEES	10,586	2–36	<0.001	0.370
EORTC_QLQ-C30	4035	2–36	0.026	0.183
DAS_Dyadic consensus	1000	2–10	0.402	0.167
DAS_Dyadic satisfaction	4882	2–36	0.013	0.213
DAS_Dyadic cohesion	7772	1530–27,536	0.004	0.302
DAS_Affectional expression	1064	1021–9185	0.330	0.106
SELF EFFICACY	1815	2–36	0.177	0.092
COPE_Social support	30,180	2–36	<0.001	0.626
COPE_Avoidance strategies	25,923	2–36	<0.001	0.590
COPE_Positive attitude	29,294	2–36	<0.001	0.619
COPE_Problem solving	28,382	2–36	<0.001	0.612
COPE_Turning to religion	53,867	2–36	<0.001	0.750
TAS-20_Difficulty Describing Feelings	3050	1170–21,063	0.060	0.145
TAS-20_Difficulty Identifying Feeling	7226	2–36	0.002	0.286
TAS-20_Externally-Oriented Thinking	6905	2–36	0.003	0.277
CORE_Wellbeing	21,327	2–36	<0.001	0.542
CORE_Problems	8720	1291–23,246	0.004	0.326
CORE_Functioning	10,987	2–36	<0.001	0.379
CORE_Risk	16,011	1401–25,224	<0.001	0.471
CORE_Total	19,877	2–36	<0.001	0.525
CORE_Total_Risk	12,166	1460–26,284	<0.001	0.403
CORE_Clinical Score	14,961	2–36	<0.001	0.454

## Data Availability

The data that support the findings of this study are available on request from the corresponding author. The data are not publicly available due to privacy or ethical restrictions.

## Appendix A

Following, a table with the twelve scheduled films.

**Table A1:** Scheduled films.

Original Title	Nationality	Year	Issue
Intouchables	FR	2011	Resources/Adaptability
10 giorni senza mamma	ITA	2019	Adaptability/Individual needs
Freedom writers	USA	2007	Courage/Resilience
Wonder	USA	2017	Positive attitude/Beyond the disease
La famille Bélier	FR	2014	Communication/Relationship
Inside Out	USA	2015	Emotions
The Bucket List	USA	2007	Time/Death
Seven Pounds	USA	2008	Change/Opportunity
Chocolat	USA	2000	Female projects/Prejudice
The Theory of Everything	UK	2014	Determination/Limits
Serendipity	USA	2001	Destiny/Trust
Love is all you need	USA	2012	Shame/Feel guilty

**Table A2:** Films contents.

TITLE	STORYLINE
Intouchables	The film deals with the issue of the psychological consequences of disability. Individuals with a medical condition tend to extend their difficulties to all areas of their life. The film focuses on how an assistant takes care of his client not focusing only on the physical aspects but also, and above all, on his emotional aspects, activating his hidden resources.
10 giorni senza mamma	This is a family comedy where the mother goes on a vacation and leaves the management and organization of the children’s daily life and activities to the father. Between dinners to prepare, kindergarten placement, embarrassing confidences of the oldest daughter, wild games with the son’s friends, quarrels, near disasters, and missed appointments at work, this film highlights the father’s initial disorganization and the subsequent implementation of functional and useful strategies, focusing attention on the ability to delegate (required by many patients in the course of therapies).
Freedom writers	This film highlights the courage of teacher Erin Gruwell as she fights for her students, motivating them to engage more and more in their studies. Through writing, he invites her students to explore and share their emotions and thoughts. The aim is not to restore order and discipline, but to seek new balances in a moment of fragility.
Wonder	The film stars a child (August) suffering from a rare genetic disease called Treacher Collins Syndrome. The film presents various topics from bullying to the importance of family support, which allows the child to regulate emotional states of sadness, anger, and self-depreciation. At the end of the film, we see how August has attracted the people around him and how diversity and pain are elements of wealth to fight prejudice, prevarication, indifference, and social isolation.
La famille Bélier	The film explores profound themes concerning diversity, growth, family life, and the communication of one’s needs. Except for one person, all family members are deaf, and this aspect highlights the inability to assume the perspective of the other.
Inside Out	This is an animated film that takes its cue from the theories of Ekman and Plutchick who theorized on primary emotions such as sadness, joy, fear, disgust, anger, and surprise that mixed together give life to secondary emotions such as love, fear, apprehension, etc. At the heart of the film, there is the value of emotions that help individuals understand who they are and why they engage in certain behaviors. All emotions are essential to ensure psychological well-being, they contribute to the formation of the personality.
The Bucket List	The film tells the story of two men with terminal cancer who decide to draw up a list of activities to be carried out. Despite the differences between the two protagonists, they agree in making a list of experiences they want to live and share in the time they have available.
Seven Pounds	A film that tells the story of an engineer played by Will Smith who causes the death of seven people including his partner, feeling terribly guilty. From that moment on, he totally changes his life and aims to save seven “good people” to alleviate his pain and guilt.
Chocolat	The film deals with the arrival of a woman with her child in a village in the French countryside and how they integrate into the small town by opening a small chocolate shop. Shortly after their arrival, the first gossip begins to spread and, consequently, the first prejudices. In the film, the protagonist’s behavior is not one of victimhood but a way to obtain a social redemption provoked by the group.
The Theory of Everything	This is a biopic about Stephen Hawking, his professional and personal history. During his illness, the character remains lucid and manages to benefit from the love of his wife and her children. The moment of diagnosis triggers fear and anger in him. At his side is always present his wife Jane, who leaves everything to devote herself completely to him and to take care of the children.
Serendipity	It is the love story of two boys who meet in front of a stall and let fate decide their next encounter. Years go by and they are both in the process of getting married until they meet again. The central theme of the film is the relationship between the two protagonists and their way of communicating and welcoming their feelings.
Love is all you need	It is the last film that the patients have seen, it is a romantic comedy where the protagonist lets her emotional suffering emerge after her husband leaves her for a younger woman, alternating it with notes of irony.

## Appendix B. Filmography

- Intouchables, Olivier Nakache, Éric Toledano (France, 2011).
- 10 giorni senza mamma, Alessandro Genovesi, Maurizio Totti e Alessandro Usai (Italia, 2019).
- Freedom writers, Richard LaGravenese, (USA, 2007).
- Wonder, Stephen Chbosky, David Hoberman, Todd Lieberman (USA, 2017).
- La famille Bélier, Éric Lartigau, Philippe Rousselet, Éric Jehelmann, Stéphanie Bermann, (France, 2014).
- Inside Out, Pete Docter, Ronnie del Carmen, Jonas Rivera (USA, 2015).
- The Bucket List, Rob Reiner, Alan Greisman, Craig Zadan, Neil Meron, producers, Rob Reiner (USA, 2007).
- Seven Pounds, Gabriele Muccino, Todd Black, Jason Blumenthal, James Lassiter, Will Smith, Steve Tisch (USA, 2008).
- Chocolat, Lasse Hallström, David Brown, Kit Golden, Leslie Holleran (USA, 2000).
- The Theory of Everything, James Marsh, Tim Bevan, Eric Fellner, Lisa Bruce, Anthony McCarten (USA, 2014).
- Serendipity, Peter Chelsom, Simon Fields, Robert Levy (USA, 2001).
- Love is all you need, Susanne Bier, Sisse Graum Jørgensen, Vibeke Windelov (USA, 2012).
